# *Helicobacter pylori*-Mediated Injury: The Hidden Path to Gastric Hemorrhage and Neoplasia

**DOI:** 10.3390/microorganisms13102392

**Published:** 2025-10-18

**Authors:** Sabrina-Nicoleta Munteanu, Ana-Maria Filip, Patrick-Lazăr-Dominik Chiciudean, Monica Pantea, Simona Mocan, Anca Elena Negovan

**Affiliations:** 1 Department of Clinical Science-Internal Medicine, “George Emil Palade” University of Medicine, Pharmacy, Science, and Technology, 540139 Târgu Mures, Romania; sabrina.munteanu@umfst.ro (S.-N.M.); cmonicapantea@gmail.com (M.P.); ancanegovan@yahoo.com (A.E.N.); 2 Oncology Department, Mureș County Clinical Hospital, 540140 Târgu Mureș, Romania; patrickcld@icloud.com; 3 Pathology Department, Emergency County Hospital of Târgu Mureș, 540136 Târgu Mureș, Romania; slmocan70@gmail.com

**Keywords:** *H. pylori* gastritis, gastric mucosal injury, gastric cancer

## Abstract

*Helicobacter pylori* infection represents a well-established risk factor for the development of gastric carcinogenesis, yet reliable clinical or endoscopic predictors of infection remain poorly defined. Identifying non-invasive or endoscopic markers of this infection could improve early detection, which is crucial for effective prevention and clinical management. This single-center study included 737 patients who underwent upper gastrointestinal endoscopy. We compared clinical, laboratory, and endoscopic features between *H. pylori*-positive and *H. pylori*-negative individuals. A total of 263 with *H. pylori*-positive gastric biopsies and 474 with *H. pylori*-negative biopsies were enrolled in our study. Cerebrovascular disease (9.51% vs. 5.51%, *p* = 0.04, OR = 1.80), type 2 diabetes mellitus (T2DM—22.05% vs. 15.86%, *p* = 0.04, OR 1.5), and alcohol consumption (18.96% vs. 9.3%, *p* = 0.00, OR = 2.28) were significantly more prevalent among *H. pylori*-positive patients. Heartburn was more commonly reported in *H. pylori*-negative individuals (23.77% vs. 15.38%, *p* = 0.01, OR = 0.58). Laboratory parameters showed no significant differences between groups. Regarding endoscopic findings, corporal erythema (26.92% vs. 16.17%, *p* = 0.00, OR = 1.91), corporal erosions (11.54% vs. 5.32%, *p* = 0.00, OR = 2.32), and submucosal hemorrhages (20.91% vs. 11.6%, *p* = 0.00, OR = 2.01) were associated with *H. pylori* infection. In the multivariate logistic regression models, alcohol consumption and corporal lesions remained independent predictors of *H. pylori*-associated gastritis, even after adjusting for age, sex, and PPI use. This study identifies alcohol consumption and specific corporal mucosal changes as novel, independent predictors of *H. pylori* infection. Heartburn was negatively associated with active *H. pylori* infection, while the rest of the symptoms did not predict infection or mucosal lesions. The laboratory parameters did not differ significantly between groups. These findings underscore the potential of targeted endoscopic evaluation and risk-based screening (particularly among T2DM and alcohol-consuming populations) to enhance early detection and management of *H. pylori*-associated disease.

## 1. Introduction

*Helicobacter pylori* (*H. pylori*) infection is associated with various gastric conditions, including chronic active gastritis, peptic ulcer, and gastric cancer. Even though the global prevalence of *H. pylori* infection declined in the past 30 years (currently estimated to be 35.1%), it still represents one of the most important carcinogenic agents [1,2].

The clinical manifestations associated with *H. pylori* infection are variable, often including dyspeptic symptoms such as epigastric pain, heartburn, bloating, and nausea/vomiting [3]. However, the majority of infected patients remain asymptomatic [4], leading to late diagnosis and treatment of this infection [5,6].

Recent studies using advances in microbiome and single-cell analyses have indicated that *H. pylori* infection leads to marked alterations in the gastric microbiota (leading to a reduction in richness and diversity) [7], along with changes in metabolic and immune pathways [8,9]. Histopathological analysis of *H. pylori*-colonized mucosa often reveals persistent inflammatory infiltrates (especially neutrophils, which represent an indicator of activity, along with lymphocytes and plasma cells in the lamina propria), intestinal metaplasia, and glandular atrophy [10,11,12]. The development of premalignant gastric lesions (intestinal metaplasia and glandular atrophy) from chronic active gastritis is closely associated with *H. pylori* infection, even in asymptomatic patients.

The interplay between clinical manifestations, endoscopic and histopathological changes, demonstrates the complex mechanisms of *H. pylori*-associated diseases. However, the clinical and endoscopic predictors that reliably indicate active *H. pylori* infection remain poorly defined. The correlation between the severity of symptoms and endoscopic changes can influence the therapeutic strategy, leading to more targeted approaches [13].

Previous studies have predominantly focused on histopathological and molecular mechanisms, leaving a critical knowledge gap regarding how routine clinical, laboratory, and endoscopic findings correlate with *H. pylori* infection status in real-world diagnostic settings. This gap limits clinicians’ ability to risk-stratify patients and optimize diagnostic strategies during the initial evaluation. Understanding these relationships is crucial for improving the management of gastric diseases in high-prevalence populations, where early diagnosis and eradication therapy are essential to prevent serious complications such as gastric cancer [14,15].

## 2. Materials and Methods

### 2.1. Selection of Patients

This single-center retrospective analysis was conducted on 737 patients who were admitted to the Emergency County Hospital of Targu Mures, Romania—Internal Medicine Clinic No. 2 between 2017 and 2024 and underwent usual blood tests and upper gastrointestinal endoscopy and biopsy prelevation.

These patients were hospitalized for various medical conditions (including cardiovascular, digestive, or renal diseases). However, upper digestive endoscopy was performed in patients with dyspeptic symptoms (epigastric pain, pyrosis, bloating, nausea/vomiting) or alarming features (loss of appetite, unexplained weight loss or anemia).

The median age of the patients included in the study was 65 years in both groups (IQR 56–73 years in *H. pylori*-positive patients versus IQR 53–74 years in *H. pylori*-negative patients).

### 2.2. Patient Selection Process

From 1134 patients who initially underwent endoscopy, 294 patients were excluded from our study based on specific criteria: *H. pylori* eradication therapy before current examination, history of cancer, irrespective of its location, end-stage heart or renal diseases, dementia, history of inflammatory bowel disease, and active bleeding (positive fecal occult test or colonic mucosal changes). Alcohol consumption was evaluated based on the frequency of intake. Individuals who reported consuming at least one unit of alcohol on three or more occasions per week (minimum of three units per week) were classified as alcohol consumers.

**Figure 1:**
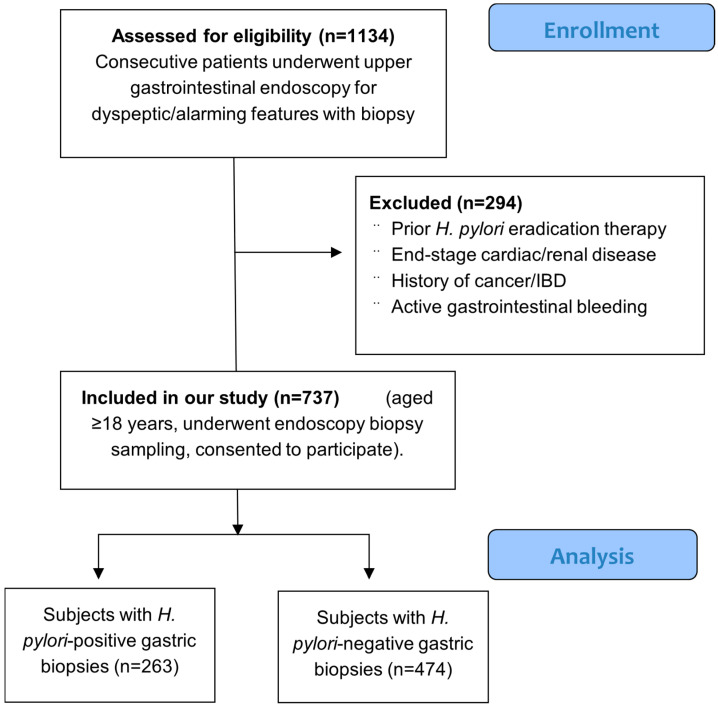
Study population selection. Abbreviations: IBD, inflammatory bowel disease.

The final study population consisted of patients 18 years and older who underwent upper endoscopy for alarming or dyspeptic features with biopsy prelevation and consented to participate in our study (Figure 1).

### 2.3. Data Collection

Patient data was collected through direct interviews and medical record review, and it included chronic medication use, particularly of those that were potentially harmful to gastric mucosa, such as anti-inflammatory drugs (NSAIDs), antiplatelet drugs (aspirin, clopidogrel, and ticagrelor), and anticoagulants (direct oral anticoagulants or antivitamin K), provided these were used for at least one month before examination. To evaluate the severity of dyspeptic symptoms, a modified version of the Leeds Dyspepsia Questionnaire was used.

Medical conditions were recorded as follows: cardiovascular diseases (valvulopathies, ischemic heart disease, and congestive heart failure), chronic kidney disease, cerebrovascular diseases (dementia, hemorrhagic or ischemic stroke), respiratory diseases (pulmonary fibrosis, asthma, and chronic obstructive pulmonary disease) and osteoarticular diseases.

Regarding laboratory values, the following normal ranges were taken into consideration (according to the reference ranges used in our hospital): hemoglobin (12–15 g/dL for women, 13–17 g/dL for men), mean corpuscular volume (MCV—80–100 fL), serum iron (11.6–31.4 µmol/L), total cholesterol (<5.17 mmol/L), triglycerides (below 1.7 mmol/L), fibrinogen (2–4.0 g/L), blood glucose (3.9–6.1 mmol/L) and INR (0.8–1.1). Statistical analyses were performed using GraphPad Prism software, version 8.4.3 (GraphPad Software, Inc., 7825 Fay Ave, Ste 230, La Jolla, CA 92037, USA).

The approval of the Ethics Committee of the Emergency County Hospital of Targu Mures, Romania, was obtained (No. Ad. 32129/27.11.2023).

### 2.4. Endoscopic and Histologic Features

The presence of gastric erosions and ulcers was documented during upper gastroscopy.

According to Sydney’s protocol, five gastric biopsies were obtained: two samples from the antrum, two from the corpus, and one from the angular notch. Each biopsy site’s samples were placed in separate containers for individual analysis [16].

Gastritis classification and grading were performed according to the Updated Sydney System protocol. Multiple staining techniques (including Hematoxylin and Eosin (H&E), modified Giemsa, and PAS-alcian blue staining methods) were used. Reactive gastropathy was diagnosed when the following histological features were present: mucin depletion in foveolar cells, foveolar hyperplasia, replacement of the lamina propria with fibromuscular tissue, and/or capillary congestion.

*H. pylori* gastritis was identified when the bacteria were observed in at least one biopsy sample using H&E and Giemsa staining techniques. In cases with persistent inflammation and proton pump inhibitor (PPI) therapy, immunohistochemistry was performed using FLEX polyclonal rabbit anti-*H. pylori* antibody (Ready to Use, DakoAutostainer (Dako Denmark A/S-Produktionsvej 42, 2600 Glostrup, Denmark) for enhanced detection [17].

Non-infectious chronic gastritis was diagnosed in the absence of *H. pylori* infection if chronic mucosal changes, lamina propria fibrosis, vascular ectasia, and varying degrees and types of inflammatory cell infiltration (including plasma cells, lymphocytes, and/or neutrophils) were present. This diagnosis required the absence of *H. pylori* infection, no history of previous eradication therapy, and no other documented infectious causes. Premalignant gastric lesions were defined as the presence of atrophic gastritis and intestinal metaplasia [18].

## 3. Results

### 3.1. Demographic Data, Comorbidities, and Behavioral Features

Enrolled patients were further stratified according to the *H. pylori* status: 263 patients with *H. pylori*-positive gastric biopsies and 474 with *H. pylori*-negative biopsies.

Regarding gender distribution, most of the study participants were females (56.49% *H. pylori*-positive females, and 60.13% *H. pylori*-negative females), without any statistically significant association between sex and *H. pylori* infection being observed (OR = 0.86, 95% CI: 0.63–1.16, *p* = 0.3487).

More than half of patients were older than 65 years (52.5% infected patients versus 56.1% uninfected patients), but the difference was not statistically significant (OR = 0.86, 95% CI: 0.64–1.16, *p* = 0.3545).

Alcohol consumption prevalence was higher among *H. pylori*-positive subjects (18.96% vs. 9.30%). A statistically significant association was identified (*p* = 0.0012, OR = 2.28, 95% CI: 1.40–3.75). Smoking showed no significant association with this infection (OR = 1.05, 95% CI: 0.68–1.61, *p* = 0.825), suggesting it does not represent a significant risk factor for *H. pylori* colonization.

Regarding comorbidities, there were no statistically significant associations between the *H. pylori* infections and cardiovascular, respiratory, renal, liver diseases or history of anemia (all *p* > 0.05). However, diabetes mellitus demonstrated a higher prevalence among *H. pylori*-positive patients (22.05% vs. 15.86%). Diabetic patients are 1.5 times more likely to develop this infection (OR 1.5, 95% CI: 1.01–2.18, *p* = 0.0453). Cerebrovascular disease was also associated with *H. pylori* colonization, affected patients showing 1.8 times higher odds of *H. pylori* infection (OR 1.80, 95% CI: 1.02–3.15, *p* = 0.0488) (Table 1).

### 3.2. Medication Use

When analyzing medication use, no significant differences were observed regarding anti-vitamin K anticoagulants (7.60% in *H.* pylori-positive vs. 9.32% in *H. pylori*-negative; OR = 0.80, 95% CI: 0.47–1.39, *p* = 0.4958). However, a statistically significant difference in *H. pylori* infection rates was observed between NOAC users and non-users (12.93% and 7.01%, respectively), with those receiving NOACs presenting almost twice the odds of developing the infection (OR = 1.97, 95% CI: 1.19–3.27, *p* = 0.0106).

The use of clopidogrel was similar between groups (12.17% vs. 12.31%), showing no significant association (OR = 0.99, 95% CI: 0.61–1.55, *p* > 0.9999). Aspirin use was more frequent among *H. pylori*-positive patients (27.00% vs. 22.51%) without reaching statistical significance (OR = 1.27, 95% CI: 0.89–1.79, *p* = 0.1781).

Surprisingly, non-steroidal anti-inflammatory drug consumption was lower in *H. pylori*-positive individuals (12.55% compared to 18.68%), reflecting a reduced probability of infection among those taking NSAIDs (OR = 0.62, 95% CI: 0.41–0.96, *p* = 0.0377).

No significant associations were found between *H. pylori* infection and the use of proton pump inhibitors, ACE inhibitors, or beta-blockers (all *p* > 0.05) (Table 2).

### 3.3. Clinical Features

For most symptoms, comparable frequencies were identified between groups: epigastric pain occurred in 39.68% of *H. pylori*-positive patients versus 46.64% of negative patients (OR = 0.75, 95% CI: 0.54–1.03, *p* = 0.0795), bloating in 14.57% versus 19.06%, respectively (OR = 0.72, 95% CI: 0.47–1.10, *p* = 0.1447), nausea or vomiting in 20.65% versus 19.96% (OR = 1.04, 95% CI: 0.71–1.52, *p* = 0.8438), loss of appetite in 12.55% versus 12.78% (OR = 0.97, 95% CI: 0.62–1.55, *p* > 0.9999), and weight loss in 14.98% versus 12.11% (OR = 1.27, 95% CI: 0.82–1.98, *p* = 0.2923).

However, heartburn demonstrated a statistically significant difference, occurring less frequently in *H. pylori*-positive patients (15.38%) compared to negative patients (23.77%; OR = 0.58, 95% CI: 0.39–0.87, *p* = 0.0109), suggesting an inverse association between *H. pylori* infection and heartburn occurrence (Table 3).

### 3.4. Endoscopic and Histopathologic Changes

In the following section, we compared endoscopic and histopathologic differences between *H. pylori*-positive and negative patients.

Of the 263 patients, only 13 (4.9%) presented with antral gastritis, while the majority demonstrated pangastritis.

The presence of *H. pylori* was associated with an increased prevalence of gastric corporal lesions, including corporal erythema (26.92% vs. 16.17%, OR = 1.91, 95% CI: 1.31–2.76, *p* = 0.0007) and corporal erosive gastritis (11.54% vs. 5.32%, OR = 2.32, 95% CI: 1.32–3.98, *p* = 0.0032), indicating that the infection has extended beyond the antrum, involving the corporal region. Antral erythema (76.1% vs. 77.2%, OR = 0.93, 95% CI: 0.65–1.35, *p* = 0.7835) and antral erosive gastritis (31.54% vs. 32.13%, OR = 0.97, 95% CI: 0.67–1.34, *p* = 0.9339) have displayed comparable frequencies among infected and non-infected patients. No significant differences were identified regarding gastric ulcer (6.46% vs. 5.70%, OR = 1.14, 95% CI: 0.61–2.17, *p* = 0.7459). However, submucosal hemorrhages (irrespective of their location) were more prevalent in infected patients (20.91% vs. 11.60%, OR = 2.01, 95% CI: 1.32–3.05, *p* = 0.0011).

Histopathologic findings were similarly distributed between groups, with atrophic gastritis occurring in 27.38% versus 26.44% of patients (OR = 1.04, 95% CI: 0.74–1.47, *p* = 0.7945) and intestinal metaplasia in 38.42% versus 33.48% (OR = 1.24, 95% CI: 0.88–1.75, *p* = 0.2189) (Table 4).

### 3.5. Laboratory Findings

No statistically significant differences were found between *H. pylori*-positive and *H. pylori*-negative patients’ laboratory parameters (Table 5). However, hemoglobin (12.7 g/dL vs. 13) and mean corpuscular volume (85.7 fl vs. 86.4 fl) values were lower in infected subjects but not statistically significant (*p* = 0.6127 and *p* = 0.3256, respectively). Similarly, *H. pylori*-positive subjects presented lower serum iron concentrations (12 µmol/L vs. 13 µmol/L), although this difference was not significant (*p* = 0.2791).

Coagulation and inflammatory markers, such as international normalized ratio (1.06 vs. 1.07) and fibrinogen (3.62 vs. 3.5), displayed comparable levels across groups (*p* > 0.05). Similarly, there were no significant differences between the glucose levels (5.52 vs. 5.4).

Cholesterol values were slightly higher in *H. pylori*-negative patients (4.36 vs. 4.63), even though the threshold of statistical significance was not reached. Triglyceride levels also showed no significant differences among groups (1.22 vs. 1.18) (Table 5).

### 3.6. Multivariate Logistic Regression Models

Model 1. Multivariate logistic regression model questioning the role of alcohol consumption and corporal lesions as independent predictors of *H. pylori* infection (unadjusted for age, sex)

In the first logistic regression analysis (unadjusted for age and sex), alcohol consumption, corporal erythema, and corporal erosive gastritis were significantly associated with *H. pylori*-positive status, being independent predictors of this infection after adjustment for PPI use. Individuals who reported alcohol use (>3 units/week) were two times more likely to be *H. pylori* positive compared with non-drinkers (OR = 2.11; 95% CI: 1.27–3.54; *p* = 0.0041). Regarding endoscopic lesions, the presence of corporal erythema (OR = 1.75; 95% CI: 1.13–2.70; *p* = 0.0114) and corporal erosive gastritis (OR = 2.29; 95% CI: 1.20–4.45; *p* = 0.013) were both significantly associated with *H. pylori* infection, while cardiovascular disease and type 2 diabetes mellitus were not significantly associated with *H. pylori* infection in the unadjusted model (Table 6).

Model 2. Multivariate logistic regression model questioning the role of alcohol consumption and corporal lesions as independent predictors of *H. pylori* infection (adjusted for age, sex, and PPI use).

In the second multivariate logistic regression model (adjusted for age, sex, and proton pump inhibitor use), alcohol consumption (OR = 2.10; 95% CI: 1.26–3.51; *p* = 0.0046), corporal erythema (OR = 1.75; 95% CI: 1.13–2.70; *p* = 0.0118), and corporal erosive gastritis (OR = 2.24; 95% CI: 1.17–4.37; *p* = 0.0158) were independently associated with *H. pylori* infection. These findings indicate that, even after adjusting for potential confounding factors, individuals who consumed alcohol (>3 units/week) or presented corporal lesions were significantly more likely to be *H. pylori* positive. Cardiovascular disease and type 2 diabetes mellitus were no longer predictors of this infection (Table 7).

Model 3. Multivariate logistic regression model questioning the role of endoscopic lesions as independent predictors of *H. pylori* infection (after adjusting for NOACs and NSAIDs use).

In the third multivariate logistic regression model (Table 8), *Helicobacter pylori* infection was significantly associated with corporal erythema and corporal erosive gastritis (after adjusting for NOACs and NSAIDs use). Participants presenting corporal erythema were approximately 1.7 times more likely to be *H. pylori* positive (adjusted OR = 1.69; 95% CI: 1.15–2.47; *p* = 0.007), while those with corporal erosive gastritis were 2 times more likely to be infected (adjusted OR = 2.01; 95% CI: 1.13–3.59; *p* = 0.017). In contrast, PPI use, NSAID use, and endoscopic evidence of gastric submucosal hemorrhage (irrespective of its location) were not significantly associated with *H. pylori* infection in this model. NOAC use was associated with *H. pylori* infection, even though it is not directly related to the development of these endoscopic lesions, but rather may increase the risk of bleeding from pre-existing gastric mucosal lesions (adjusted OR = 1.86; 95% CI: 1.10–3.13; *p* = 0.020).

### 3.7. Spearman Correlation Between H. pylori Status and Laboratory Parameters

The Spearman correlation analysis did not identify any significant relationships between *H. pylori* infection status and any of the measured laboratory parameters (hemoglobin, MCV, serum iron, fibrinogen, INR, total cholesterol, glucose, and triglycerides; all *p* > 0.05). The correlation coefficients were close to zero, suggesting that these hematological and biochemical parameters are not influenced by the *H. pylori* status, therefore not presenting predictive value for infection in this population (Table 9).

## 4. Discussion

In the present study, we performed an analysis of the social, demographic, clinical, endoscopic, and histopathologic factors associated with *H. pylori* infection in a relatively small population. In alignment with recent studies [15,19] indicating that sex differences in *H. pylori* infection rates are relatively small across groups and may be influenced by various sociodemographic factors, the stratification of patients by *H. pylori* status did not reveal a statistically significant association between sex and infection. However, some studies indicate that men were more often affected by this infection [20,21].

Alcohol consumption was associated with a higher risk of *H. pylori* infection identified (*p* = 0.0012, OR = 2.28, 95% CI: 1.40–3.75) and continued to be an independent predictor for this infection in the multivariate logistic regression model even after adjusting for age, sex and PPI use (OR = 2.10; 95% CI: 1.26–3.51; *p* = 0.0046). Previous research presents conflicting findings on alcohol’s relationship with *H. pylori* infection. Several studies demonstrate a protective effect of moderate alcohol consumption against *H. pylori* [22,23,24,25], especially in patients who drank wine or mixed types of alcoholic drinks [26], while others report increased prevalence rates [27]. These results seem to be influenced by population-specific factors, drinking habits, and potential confounders. However, in our study, subjects consuming more than three alcohol units per week had approximately twice the odds of *H. pylori* infection compared with non-drinkers, supporting the hypothesis that alcohol-induced mucosal injury may facilitate bacterial adhesion or persistence.

Cerebrovascular disease (including history of stroke and dementia) seems to be associated with a higher chance of *H. pylori* colonization (OR 1.80, 95% CI: 1.02–3.15, *p* = 0.0488), probably due to atherothrombosis and small artery disease [28,29]. Recent data suggest that cardiovascular disease risk was slightly elevated in *H. pylori*-infected patients, though this association did not extend to stroke risk, which remained non-significant [30].

Diabetic patients are 1.5 times more likely to develop this infection (OR 1.5, 95% CI: 1.01–2.18, *p* = 0.0453). However, it did not remain an independent predictor of the infection in the multivariate regression models, whether adjusted or unadjusted for age and sex. The apparent link between these entities might reflect the underlying chronic systemic inflammation, which can promote insulin resistance [31,32] and probably gut microbiota alteration (with a significant reduction in *Lactobacillus*, *Akkermansia*, and *Butyricimonas* genera in the fecal samples of *H. pylori*-positive patients) [33]. In order to better establish this association, HbA1c measurements would be necessary.

*H. pylori* infection was significantly associated with increased use of NOACs, with users showing nearly double the odds of developing this infection compared to non-users (12.93% vs. 7.01%, OR = 1.97, *p* = 0.0106). NOAC use was associated with *H. pylori* infection, even though it is not directly related to the development of these endoscopic lesions, but rather may increase the risk of bleeding from pre-existing gastric mucosal lesions and delayed healing (adjusted OR = 1.86; 95% CI: 1.10–3.13; *p* = 0.020) [34].

Surprisingly, NSAIDs use was associated with a lower probability of infection due to a decreased use rate in *H. pylori*-positive patients, who reported significantly less NSAID utilization than *H. pylori*-negative individuals (12.55% vs. 18.68%, OR = 0.62, *p* = 0.0377). In the multivariate regression model, it was not an independent predictor of this infection. The interaction between these factors in causing erosive gastritis or corporal erythema remains controversial, with no clear evidence of a significant synergistic effect [35,36].

Regarding clinical manifestations, the prevalence of dyspeptic and alarming features was comparable in *H. pylori* across groups, suggesting that specific gastrointestinal symptoms cannot reliably differentiate between infected and uninfected patients, consistent with previous literature findings [6,37,38,39].

Regarding heartburn (23.77%; OR = 0.58, 95% CI: 0.39–0.87, *p* = 0.0109), multiple studies have identified an inverse relationship between *H. pylori* infection and the occurrence of heartburn and gastroesophageal reflux disease symptoms [40,41].

Corporal lesions were significantly more prevalent in *H. pylori*-infected patients, with corporal erythema occurring in 26.92% versus 16.17% of uninfected patients (OR = 1.91, 95% CI: 1.31–2.76, *p* = 0.0007) and corporal erosive gastritis in 11.54% versus 5.32% (OR = 2.32, 95% CI: 1.32–3.98, *p* = 0.0032). Even after adjusting for age, sex, and proton pump inhibitor use, corporal erythema (OR = 1.75; 95% CI: 1.13–2.70; *p* = 0.0118) and corporal erosive gastritis (OR = 2.24; 95% CI: 1.17–4.37; *p* = 0.0158) were independent predictors of *H. pylori* infection. The severity of gastritis correlates with the density of *H. pylori* colonization. However, the predictive value of endoscopic findings alone for *H. pylori* infection is modest, and histopathological confirmation remains essential [42,43].

Prior studies have reported corporal involvement in *H. pylori*-infected patients, indicating that *H. pylori* colonization has progressed beyond the antrum to involve the gastric corpus. The *H. pylori* colonization was pangastric in younger patients, or it affected predominantly the corpus in older groups, extending with increasing age [44,45].

Pangastritis occurred in most of our patients (96.1%), while antral gastritis was observed in only 13 patients (4.9%). This pangastritis phenotype is likely determined by several factors, including Romania’s high *H. pylori* prevalence [46,47] and the advanced age of our group, with more than half of the patients being over 65 years). A recent study from Romania reported an increased frequency of corporal metaplasia and inflammation, which was correlated with altered lipid profiles and persistent chronic inflammation, suggesting a link between *H. pylori* and metabolic disturbances, including dyslipidemia and features of metabolic syndrome [46].

This distal-to-proximal progression pattern is often observed in elderly patients, where both the severity of colonization and the degree of corporal inflammation increase significantly with age [48,49].

Surprisingly, premalignant gastric lesion distribution was similar between groups, with atrophic gastritis occurring in 27.38% versus 26.44% of patients (OR = 1.04, 95% CI: 0.74–1.47, *p* = 0.7945) and intestinal metaplasia in 38.42% versus 33.48% (OR = 1.24, 95% CI: 0.88–1.75, *p* = 0.2189). These similar rates might occur due to prior *H. pylori* infection in patients who currently test negative for this infection, yet present significant atrophic and metaplastic changes. Unintentional clearance of *H. pylori* infection in patients infected during childhood can occur as a result of exposure to antibiotics prescribed for other indications, particularly if the antibiotics have activity against *H. pylori*, such as amoxicillin, clarithromycin, or metronidazole. This phenomenon is often seen in patients with atrophic gastritis and no history of intentional eradication therapy, where *H. pylori* is no longer detectable [50].

Patients with chronic atrophic gastritis testing negative for active *H. pylori* without documented eradication treatment have been recognized, emphasizing the necessity for systematic endoscopic surveillance [50]. However, differential diagnosis must exclude autoimmune gastritis and consider potential false-negative testing results.

We did not systematically exclude autoimmune gastritis, which may represent a confounding factor in the evaluation of gastric lesions. Furthermore, using immunohistochemical techniques for *H. pylori* identification is required, given their superiority over standard histological examination in bacterial detection accuracy. A strength of our study is that *H. pylori* detection was based on histopathological examination, including an immunohistochemistry test in case of severe inflammation without *H. pylori* presence on basic coloration. This may have excluded a risk of false-negative results, particularly in patients with prior proton pump inhibitor use.

Concerning laboratory parameters, hemoglobin (12.7 g/dL vs. 13), mean corpuscular volume (85.7 fl vs. 86.4 fl) and serum iron concentrations (12 µmol/L vs. 13 µmol/L) values were lower, although the differences were not statistically significant (all *p* > 0.05).

The relationship between anemia and *H. pylori* colonization is well known [51,52], even though some studies did not identify an impact of *H. pylori* infection on hematological parameters [53]. The lack of significant differences might be caused by the multifactorial nature of anemia and iron deficiency and prior spontaneous eradications.

In our study, cholesterol values were slightly higher in *H. pylori*-negative patients (4.36 vs. 4.63), in contradiction with the results from recent studies that demonstrated that infected patients displayed significant changes in the serum lipid profile of infected patients [53,54,55].

In the Spearman analysis, none of the tested parameters showed significant associations with infection status (*p* > 0.05 for all comparisons). Therefore, hematological and biochemical parameters cannot serve as reliable diagnostic indicators of *H. pylori* infection and its complications in this population.

Unlike previous studies that have primarily focused on isolated clinical or histopathological characteristics, our study provides an integrated analysis combining demographic, clinical, endoscopic, and histopathological parameters in a real-world hospital setting, allowing us to identify alcohol consumption and specific corporal mucosal lesions as independent predictors of active *H. pylori* infection, even after adjustment for potential confounders.

These findings suggest that easily recognizable endoscopic features and lifestyle-related factors can help refine the clinical suspicion of infection in daily practice, especially in settings where rapid diagnostic tests are not routinely available.

Furthermore, the observed inverse association between heartburn and *H. pylori* infection reinforces the concept that symptomatic presentation alone is not a reliable diagnostic indicator, highlighting the need for targeted screening protocols rather than symptom-based testing.

By integrating clinical and endoscopic predictors, our study provides new, practice-oriented evidence that may facilitate more accurate risk stratification and improved management of *H. pylori*-related disorders, especially in populations with a high infection prevalence.

Several factors may limit the validity of these results, including the relatively small sample size, single-center enrollment, and relatively similar sociodemographic characteristics of the study population. Another limitation is represented by the inability to discontinue proton pump inhibitors before endoscopy, since many patients were admitted with comorbidities or alarming gastrointestinal features (such as anemia).

However, we performed integrated histo-endoscopic-clinical analysis, correlating endoscopic appearances with histological features (inflammation chronicity and activity, atrophy, intestinal metaplasia) and clinical parameters.

## 5. Conclusions

In our study, gastric corporal lesions and alcohol consumption were independent predictors of *H. pylori* infection. Cerebrovascular disease and type 2 diabetes mellitus were also associated with increased prevalence of this infection, emphasizing the systemic implications of chronic inflammation and metabolic dysfunction. Symptoms could not predict the presence of bacteria and inflammation in gastric biopsies. However, heartburn was negatively associated with *H. pylori* infection, consistent with the inverse relationship described previously between *H. pylori* and gastroesophageal reflux symptoms.

A higher prevalence of corporal erythema and erosive lesions was identified in infected patients, probably due to a higher frequency of the pangastritis phenotype in the studied population.

However, the occurrence of premalignant lesions did not differ significantly between these groups, suggesting prior spontaneous eradication or false-negative results during the long-term evolution of infection may account for these findings. These results highlight that *H. pylori* infections are associated with inflammatory changes in gastric mucosa, but the lack of significant difference in the occurrence of premalignant lesions suggests that factors beyond current infection status may be influencing the prevalence of these lesions (including past exposure and genetic susceptibility).

Laboratory parameters, including hemoglobin, mean corpuscular volume, serum iron, and lipid profile, did not significantly differ by *H. pylori* status, and no significant correlations were found, indicating that hematological and biochemical markers are not reliable diagnostic indicators in this context. Regarding limitations, our study’s single-center, retrospective design may restrict generalizability, and the lack of systematic evaluation for autoimmune gastritis could confound the interpretation of gastric lesions. *H. pylori* detection relied on histopathology, without confirmation by non-invasive tests, potentially leading to false-negative results. The advanced age of our subjects and incomplete data on prior spontaneous eradication therapy may also limit broader applicability. Further validation in larger, multicenter prospective studies is required.

In conclusion, these results underscore the necessity for early detection and specific screening protocols, especially for individuals with type 2 diabetes mellitus and those with cerebrovascular disease. Larger, multicenter studies are necessary in order to validate the applicability of these results.

## Figures and Tables

**Table 1:** Differences regarding demographic data, comorbidities and behavioral features in *H. pylori*-positive and negative patients.

	*H. pylori* Positive Patients		*H. pylori* Negative Patients		*p*	OR	95% CI
**Female sex**	148	56.49%	285	60.13%	0.3487	0.86	0.63–1.16
**Lifestyle**							
Smoking	40	17.62%	65	16.88%	0.825	1.05	0.68–1.61
**Alcohol**	**40**	**18.96%**	**33**	**9.30%**	**0.0012**	**2.28**	**1.40–3.75**
**Comorbidities**							
Cardiovascular disease	197	74.90%	340	71.73%	0.3875	1.17	0.83–1.66
Liver disease	61	23.19%	96	20.25%	0.3496	1.18	0.81–1.69
Respiratory disease	52	19.77%	94	19.83	>0.9999	0.99	0.68–1.45
History of anemia	79	30.04%	149	31.50%	0.7394	0.93	0.67–1.29
**Type 2 Diabetes** **mellitus**	**58**	**22.05%**	**75**	**15.86%**	**0.0453**	**1.50**	**1.01–2.18**
**Cerebrovascular** **disease**	**25**	**9.51%**	**26**	**5.51%**	**0.0488**	**1.80**	**1.02–3.15**
Chronic kidney disease	33	12.55%	63	13.32%	0.8198	0.93	0.59–1.47

**Table 2:** Drug use among *H. pylori*-positive and negative patients.

Drugs	*H. pylori* Positive Patients		*H. pylori* Negative Patients		*p*	OR	95% CI
Anti-vitamin K	**20**	7.60%	44	9.32%	0.4958	0.80	0.47–1.39
**NOAC**	**34**	**12.93%**	**33**	**7.01%**	**0.0106**	**1.97**	1.19–3.27
Clopidrogel	32	12.17%	58	12.31%	>0.9999	0.99	0.61–1.55
Aspirin	71	27.00%	106	22.51%	0.1781	1.27	0.89–1.79
**NSAIDs**	**33**	**12.55%**	**88**	**18.68%**	**0.0377**	**0.62**	**0.41–0.96**
PPI	150	57.03%	278	59.02%	0.6396	0.92	0.68–1.25
ACEI	100	38.02%	150	31.85%	0.1042	1.31	0.96–1.79
Beta-blockers	129	49.05%	230	48.83%	>0.9999	1.01	0.75–1.36

Abbreviations: NOAC—Non-Vitamin K Oral Anticoagulants; NSAIDs—Nonsteroidal Anti-Inflammatory Drugs; PPI—Proton Pump Inhibitors; ACEI—Angiotensin-Converting Enzyme Inhibitors.

**Table 3:** Distribution of clinical features in *H. pylori*-positive and negative patients.

	*H. pylori* Positive Patients		*H. pylori* Negative Patients		*p*	OR	95% CI
**Symptoms**							
Epigastric pain	98	39.68%		46.64%	0.0795	0.75	0.54–1.03
**Heartburn**	**38**	**15.38%**	**106**	**23.77%**	**0.0109**	**0.58**	**0.39–0.87**
Bloating	36	14.57%	85	19.06%	0.1447	0.72	0.47–1.10
Nausea/vomiting	51	20.65%	89	19.96%	0.8438	1.04	0.71–1.52
Loss of appetite	31	12.55%	57	12.78%	>0.9999	0.97	0.62–1.55
Weight loss	37	14.98%	54	12.11%	0.2923	1.27	0.82–1.98

**Table 4:** Group differences regarding endoscopic and histopathologic features in *H. pylori*-positive and negative patients.

	*H. pylori* Positive Patients		*H. pylori* Negative Patients		*p*	OR	95% CI
**Endoscopic Changes’**							
Antral erythema	198	76.1%	363	77.2%	0.7835	0.94	0.65–1.35
**Corporal erythema**	**70**	**26.92%**	**76**	**16.17%**	**0.0007**	**1.91**	**1.31–2.76**
Antral erosive gastritis	82	31.54%	151	32.13%	0.9339	0.97	0.70–1.34
**Corporal erosive gastritis**	**30**	**11.54%**	**25**	**5.32%**	**0.0032**	**2.32**	**1.32–3.98**
Gastric ulcer (antral or corporal)	17	6.46%	27	5.70%	0.7459	1.14	0.61–2.17
Submucosal hemorrhage	**55**	**20.91%**	**55**	**11.60%**	**0.0011**	**2.01**	**1.32–3.05**
Duodenal lesions	130	49.43%	240	50.63%	0.759	0.95	0.70–1.28
**Histopathologic Changes**							
Atrophic gastritis	72	27.38%	124	26.44%	0.7945	1.04	0.74–1.47
Intestinal metaplasia	78	38.42%	157	33.48%	0.2189	1.24	0.88–1.75

**Table 5:** Distribution of laboratory findings among H pylori-positive and -negative patients.

Parameter	*H. pylori*-Positive Patients	*H. pylori*-Negative Patients	*p*-Value
Hemoglobin (g/dL)	12.7 (10.5–14)	13 (10.85–14.2)	0.6127
Mean corpuscular volume (fL)	85.7 (81.6–89.4)	86.4 (82.05–89.3)	0.3256
Serum Iron (µmol/L)	12 (7.61–16.65)	13 (8–17.8)	0.2791
Fibrinogen (g/dL)	3.62 (3.1–4.18)	3.5 (2.93–4.4)	0.5449
International Normalized Ratio	1.06 (0.97–1.22)	1.07 (0.98–1.27)	0.2739
Cholesterol (mmol/L)	4.36 (3.55–5.53)	4.63 (3.64–5.58)	0.3978
Triglycerides (mmol/L)	1.22 (0.88–1.79)	1.18 (0.84–1.67)	0.183
Glucose (mmol/L)	5.52 (4.87–6.25)	5.4 (4.93–6.04)	0.326

**Table 6:** Multivariate logistic regression model questioning the role of alcohol consumption and corporal lesions as independent predictors of *H. pylori* infection (unadjusted for age, sex).

Parameter	B (Estimate)	SE	*p*-Value	Adjusted OR	95% CI
**Alcohol use**	**0.748**	0.260	**0.0041**	**2.11**	**1.27–3.54**
PPI use	−0.236	0.1839	0.199	0.79	0.55–1.13
**Corporal erythema**	**0.558**	0.2208	**0.0114**	**1.75**	**1.13–2.70**
**Corporal erosive gastritis**	**0.828**	0.3335	**0.0130**	**2.29**	**1.20–4.45**
Cardiovascular disease	0.1204	0.2098	0.566	1.13	0.75–1.71
Type 2 Diabetes mellitus	0.4109	0.2467	0.096	1.51	0.93–2.45

Abbreviations: PPI–Proton Pump Inhibitors.

**Table 7:** Multivariate logistic regression model questioning the role of alcohol consumption and corporal lesions as independent predictors of *H. pylori* infection (adjusted for age, sex and PPI use).

Parameter	B (Estimate)	SE	*p*-Value	Adjusted OR	95% CI
Male sex	0.062	0.1836	0.734	1.06	0.75–1.52
Age	−0.0056	0.0075	0.454	0.99	0.98–1.01
**Alcohol use**	**0.74**	0.261	**0.0046**	**2.10**	**1.26–3.51**
PPI use	−0.236	0.184	0.208	0.79	0.51–1.13
**Corporal erythema**	**0.558**	0.2216	**0.0118**	**1.75**	**1.13–2.70**
**Corporal erosive gastritis**	**0.8081**	0.3347	**0.0158**	**2.24**	**1.17–4.37**
Cardiovascular disease	0.2226	0.2478	0.369	1.25	0.77–2.04
Type 2 Diabetes mellitus	0.4122	0.2498	0.098	1.51	0.92–2.46

Abbreviations: PPI—Proton Pump Inhibitors.

**Table 8:** Multivariate logistic regression model questioning the role of endoscopic lesions as independent predictors of *H. pylori* infection (after adjusting for NOACs and NSAIDs use).

Parameter	B (Estimate)	SE	*p*-Value	Adjusted OR	95% CI
PPI use	−0.004	0.163	0.982	1.00	0.72–1.37
**Corporal erythema**	**0.523**	0.194	**0.007**	**1.69**	**1.15–2.47**
**Corporal erosive gastritis**	**0.699**	0.292	**0.0169**	**2.01**	**1.13–3.59**
**NOAC use**	**0.618**	0.265	**0.0197**	**1.86**	**1.10–3.13**
NSAIDs use	−0.357	0.229	0.117	0.70	0.44–1.09
Submucoal hemorrhage (irrespective of location)	0.975	0.787	0.212	2.65	0.57–13.20
Antral submucosal hemorrhage	−0.791	0.718	0.275	0.45	0.10–1.83
Corporal submucosal hemorrhage	−0.054	0.728	0.942	0.95	0.21–3.90

Abbreviations: NOAC—Non-Vitamin K Oral Anticoagulants; NSAIDs—Nonsteroidal Anti-Inflammatory Drugs; PPI—Proton Pump Inhibitors.

**Table 9:** Spearman correlation between *H. pylori* status and laboratory parameters.

Laboratory Parameters	Spearman r	*p*-Value
Hemoglobin (g/dL)	0.032	0.452
Mean corpuscular volume (fL)	0.005	0.915
Serum Iron (µmol/L)	−0.056	0.222
Fibrinogen (g/dL)	0.078	0.147
INR	−0.063	0.210
Total Cholesterol (mmol/L)	−0.062	0.161
Glucose (mmol/L)	0.038	0.387
Triglycerides (mmol/L)	0.025	0.580

## Data Availability

The original contributions presented in this study are included in the article. Further inquiries can be directed to the corresponding author.

## Abbreviations

The following abbreviations are used in this manuscript:

CI	Confidence Interval
H&E	Hematoxylin and Eosin
*H. pylori*	*Helicobacter pylori*
INR	International Normalized Ratio
IQR	Interquartile Range
MCV	Mean Corpuscular Volume
NSAIDs	Non-Steroidal Anti-Inflammatory Drugs
OR	Odds Ratio
T2DM	Type 2 Diabetes Mellitus
